# Editorial

**Published:** 1862-07

**Authors:** 


### Editorial.

ARMY SURGEONS.—We receive many letters of inquiry in relation to the steps necessary to procure an appointment as surgeon or assistant-surgeon in the regiments of volunteers for the present war. For the information of all our readers, we state that it is necessary, first, to apply to the Governor of this State for permission to go before the Board of Medical Examiners for the State. Second, to pass a satisfactory examination by such Board, whose duty it is to give a certificate designating whether the candidate is recommended as an assistant-surgeon, or surgeon. Having passed the examination of the Board successfully, the candidate is elligible to appointment whenever a vacancy can be found in any of the regiments belonging to this State.

Such appointments will be much facilitated if the candidate can obtain also, a recommendation from the colonel commanding the regiment in which the vacancy exists.

We are informed that the next regular session of the Medical Board for the examination of candidates, will commence in Springfield, Illinois, on the 4th of August next.

The call for 300,000 more men for the war, makes it necessary to organize a large number of new regiments in this State, with a corresponding number of new places for surgeons and assistants.

The experience of the past year has clearly demonstrated, that *two* medical officers, namely a surgeon and one assistant, are not sufficient for a regiment of volunteers, constantly engaged in active service. The authorities of some of the States, like those of Wisconsin, foresaw the insufficiency of the regular number, and consequently sent a second-assistant-surgeon with each of their regiments. This ought to be done by all the States, certainly with the new regiments now being organized. No policy can be more short-sighted or reprehensible than that of supplying an army with an insufficient number of medical officers, or an insufficient quantity and variety of medical stores.

Will not the Board of Medical Examiners for this State, who have intercourse and influence with the Governor and other military authorities, make a strong effort to have every new regiment from Illinois supplied with at least one surgeon and *two* assistants?

We would also urge all those members of the profession, who intend to seek appointments in the army, to procure and thoroughly read those works relating especially to army hygiene; or the best means of preventing disease. Many of our young physicians commit a great mistake. They connect with army duties only the idea of operative surgery, and hence, in their preparation, are more anxious about the details of anatomy and practical surgery than anything else. Of course, these branches should not be neglected; but all experience has shown that diseases caused by bad cooking, bad camping, bad and uncleanly habits, and bad air from faulty arrangements in camps and hospitals, kill *ten* soldiers for every *one* slain in battle. Let every medical aspirant for army service study these matters with the utmost care.

---

### CLINICAL CASES IN THE MEDICAL WARDS OF THE MERCY HOSPITAL.

Organic Disease of the Heart.—Anasarca, &c.—Mrs. C., a native of Ireland, aged about 40 years, was admitted to the Hospital four days since. The case well illustrates the extent to which disease may effect changes, both in structure and function. Three or four years since she first came under my observation at her own house. She was complaining of irregular action of the heart, difficulty of breathing, scanty urine, and œdematous extremities. Direct examination of the chest by auscultation and percussion, revealed the existence of a rough bellows murmur over the region of the aortic valves, and moderate hypertrophy of the heart.

Under the influence of sedative and diuretic remedies her symptoms were so far relieved that she passed from my care for a time. After a few months all the symptoms returned, but were again relieved by similar treatment. About one year has elapsed since I last saw her until she entered the hospital a few days since. When she was brought here her breathing was short and oppressed, her face pale and bloated, the whole exterior tissues of the body and limbs œdematous, and a large effusion into the cavity of the peritoneum.

The lower extremities were enormously swollen with serous infiltration into the cellular tissues; the skin from the foot to the knee very red and abraded in several places, while the top of the left foot was occupied by an extensive gangrenous slough, partially separated, and underneath filled with "maggots." The burning, scalding pain in the feet and legs was very severe. The case is interesting, as an illustration of the extensive changes that may take place in the blood and tissues from the long continuance of a simple structural lesion primarily limited to the valves of the aorta.

The prognosis was, of course, very unfavorable; there being no hope of doing more than to palliate the more severe symptoms.

For this purpose an effort was made to increase the secretion of the kidneys so as to lessen the watery element of the blood, and thereby diminish the general dropsical effusions. The object being simply to diminish the amount of water without impairing the strength of the patient, or increasing the metamorphosis of tissues, the diuretic chosen was a mixture of nitrous ether, ℥iss, and tincture of digitals, ℥ss; of which a fluid drachm was given every three hours. The lower extremities were placed in a horizontal position and the surface, so far as it was occupied by the erythematic inflammation which occasioned so much burning pain, was covered with an ointment containing 20 grs. of acatate of lead, and 10 grs. of sulph. morphine, to the ounce of simple cerate. The gangrenous portion of the top of the foot was thoroughly washed with a solution of chloride of zinc, 2 grs. to the ounce of water, which speedily destroyed both the offensive smell and the *maggots*, and facilitated the separation of the sloughs.

Under this treatment, aided by a mild but nutritious diet, her most distressing symptoms have been much relieved; but the evident organic changes which have long existed in the valves and muscular structure of the heart, and the consequent altered properties of the solids and fluids throughout the whole system, preclude even the hope of accomplishing more than temporary relief.

---

Syphilitic Ecthyma.—Mr. B., aged about 28 years; native of Germany, was admitted to the hospital about two months since, with the surface of the nates, perinium, and one groin thickly studded with pustules of ecthyma; and the scrotum covered with an eruption of chronic eczema. These eruptions had existed several months before his admission, during which time he had been subjected to a variety of treatment with but little apparent advantage. The dark red, or coppery hue of the areola around the pustules, together with the fact that he had had primary syphilis a few years previous, rendered it quite certain that the present eruption was influenced by that disease.

The thick, conical scabs had been detached from most of the sores, making them have the appearance of ill-conditioned ulcers with elevated edges, and very painful.

But the scabs were left unbroken on a sufficient number to render the characteristic features prominent and easily recognized. There was not much general debility, and the digestive organs were in a healthy condition.

*Treatment.*—He was directed to take *ten* drops of Donovan's solution in sweetened water just before each meal, and at bedtime. To allay the pain and destroy the irritability of the ulcers, they were wet twice a day with an aqueous solution of tannin and opium; and in the interval dressed with an ointment of ammoniated mercury and simple cerate.

It was soon rendered apparent, however, that all applications in the form of ointments only aggravated the cutaneous eruption. They were consequently dispensed with, and the surface kept covered with a cloth, wet in a solution of sulphate of iron, 5 grs. to the ounce of water. A few of the larger ulcers were touched once or twice with the solid nitrate of silver. Under this treatment the eczemous eruption on the scrotum rapidly disappeared, but the improvement in the ecthymous pustules and ulcers was slow. When he had taken the Donovan's solution about two weeks, his gums and breath began to exhibit all the characteristic symptoms of mercurial action. Its use was then restricted to one dose of ten drops every night; but in a few days the mouth became so sore and the breath so offensive, that it was omitted altogether, and the following substituted in its place, viz.:—

℞. Fluid Ext. of Conium, .................... ℥jss.

Fluid Ext. of Stillingia, .................. ℥jss.

Iodide of Potassa, .......................... ʒjj.

Mix, and take a teaspoonful before each meal and at bedtime.

Under this treatment, with a continuance of the local application of the solution of sulphate of iron, the ulcers and pustules began to cicatrize more rapidly, and are now, ten weeks after admission into the Hospital, quite well.

Many have supposed that the internal use of Donovan's solution would not produce mercurial salivation. But it certainly produced that effect in the present case, after having been used only about two weeks. Whether the patient was *peculiarly* susceptible to the influence of mercurials generally, I do not know.

---

Chronic Diarrhœa.—Rubeola, &c.—A. B., an orphan boy, aged about 10 years, was presented to the Clinical class, July 21st, 1862. He was slender, thin in flesh, face and lips pale, skin cool, tongue clean, pulse moderately accelerated, soft, and small. His bowels were loose, having had, during the last four or five days, from three to six thin, brown, or greyish-brown stools per day, with loss of appetite and strength, but without much pain. Several of the lymphatic glands, behind the angle of the jaw, and along the margin of the sterno-cleido-mastoid muscle, were enlarged and slightly tender to the touch.

The patient had passed through a regular ordinary attack of rubeola or measles, commencing about two weeks previous.

There was nothing in the appearance of the present patient to mark it as one of any interest or importance; and yet the class were reminded that it was a good sample of a class of cases, of not unfrequent occurrence, and which by neglect or bad treatment sometimes lead to pathological changes of the most serious nature. Diarrhœa, depending on a low grade of irritation of the mucous membrane of the ileum and colon, is a common occurrence during the decline of the eruptive stage and the early convalescence of measles and scarlet-fever. In most cases this diarrhœa continues only a few days, and ceases spontaneously without attracting any attention; but if the patient is naturally delicate in organization, or hereditarily predisposed to scrofula, the irritation of the mucous membrane becomes more persistent.

The mesenteric glands connected with the effected portion of mucous membrane soon partake of the same grade of excitement, leading to increased vascularity and tumefaction of those glands. This increased vascularity is soon followed by the deposit of matter either plastic or ca-coplastic (scrofulous), and permanent enlargement.

This state of the glands interferes with the further absorption and assimilation of chyle, consequently the fœcal evacuations remain unhealthy, emaciation steadily progresses, and in from three to six months all the phenomena of marasmus or tabes mesenterica, are fully developed.

In persons more nearly the age of puberty, an attack of measles is often followed by the same grade of irritation in the mucous membrane of the respiratory passages, perpetuating a troublesome cough, and the slow formation of deposits, either scrofulous or tuberculous, in the bronchial glands and sometimes in the tissue of the lungs. Ultimately the patient presents all the symptons and results of ordinary consumption. Such are the very serious consequences which often result from the neglect of that simple and apparently mild grade of irritation in the mucous membranes and glands presented by the patient before us.

Hence it is a matter of much practical importance, to understand the nature and tendencies of these cases early, that appropriate treatment may be resorted to before the more serious pathological changes have taken place.

In selecting remedies for the treatment of the child before us, it is necessary to keep the objects to be accomplished clearly in the mind. These objects are: 1st, to allay the irritability or morbid sensitiveness of the mucous surfaces, both pulmonary and intestinal; 2nd, to increase the general tone of the capillaries and maintain a free action in those organs through which effete matter is rejected from the system, especially the kidneys; 3d, to diminish the frequency of the intestinal discharges.

The first and last of these objects may be accomplished by almost any combination of anodyne and astringent remedies, such as an opiate with acetate of lead, tanin, kino, or any other pure astringent.

Every observing practitioner has learned that these combinations not only allay the irritation of the mucous membranes, and lessen the discharges from the bowels, but the are liable to diminish, also, secretory action generally, and thereby lessen the elimination of urine and other effete matter to an injurious extent. Hence we prefer to combine the opiate necessary for allaying the irritation with such agents as will improve the general tone of the capillaries and increase secretion, especially from the kidneys. Either of the following formulæ will meet the indications perfectly:—

℞. Ol. Terebinth.......................... ʒjj.

Tinct. Opii.,.......................... ʒjj.

Pulv. G. Acaccia,...................... ʒjjj.

Sacchar. Alba,......................... ʒjjj.

Rub together and add

Mint Water,............................℥jj.

Mix, and give to the patient before us half a fluid drachm three times a day.

Or, ℞. Aromat. Sulph. Acid,.................. ʒjj.

Sulph. Magnesia,....................... ʒjj.

Tinct. Opii.,.......................... ʒjj.

Water,................................. ℥jj.

Mix. Dose the same as the preceding.

Or, ℞. Erigeron (Fleabane),.................. ℥j.
Tannate of Quinine,....................20 grs.
Sulph. Morph.,......................... 1 gr.

Mix, make an infusion in a pint of boiling water. When cold, give from one to two fluid drachms every three, four, or six hours according to the frequency of the discharges. These combinations are not only applicable to the treatment of such cases as the one under consideration, but we have used them for a number of years, with great satisfaction, in the treatment of the second stage of cholera morbus, cholera infantum, and the common summer complaints of children.

---

Medical Department of Lind University.—The fourth annual announcement of this institution has been laid on our table. The changes which have occurred in the faculty were duly noticed in a previous number of the Examiner. The announcement affords evidence of a very gratifying degree of prosperity on the part of this department of the university. The next regular annual lecture term commences on the second Monday in October and closes on the first Monday in March following.

There is now present an excellent class of students attending the clinics and examinations of the summer course, and the prospects for the coming term are good.

---

Two Assistant-Surgeons.—Since the first editorial article was written, we notice with much pleasure that our State authorities have directed *two* assistant-surgeons for each Illinois regiment.

---

### SELF-APPLYING SPONGE PESSARY.

To the Editor of the Lancet:—

Sir:—Though old, I am not dead yet; and, in relinquishing the practice of my profession, which I have exercised for the space of forty-four years in the metropolis—*nec ignotus, nec ignorus*—I wish to leave in your pages another record of my devotion to that branch especially which has reference to maladies afflicting the other sex.

Under the name of "Self-applying Sponge Pessary," I desire to make known a very simple contrivance by which uterine prolapsus (unless of a very aggravated form) can be effectually supported with the greatest ease, without the manual interference of a medical attendant.

Sponge pessaries have ever been in favor with the patients themselves, for many obvious reasons: amongst others, their pliability—the complete manner in which they apply themselves to the vagnia imparting a feeling of support, and further, the facility they afford of preserving cleanliness by means of injections with the improved female syringe. But if the sponge is of a large size, it is not readily inserted by the ordinary process. Now the object of my contrivance is to facilitate the latter operation by the patient herself.

That contrivance consists, first, of a conveying tube, four inches and a-half in length; and, secondly, of a slender stem, intended to bear the sponge. Both are made of the finest and lightest turned ivory, having a high polish. The diameter of the tube is one inch (or one inch and a-half if a larger sponge be required) at the upper end; and one inch, half an inch, or three-quarters of an inch, as may be, at the lower end. Here there are two semi-oval pieces on the ivory walls cut out, opposite one another, an inch high; the remaining edges of this end of the tube being slightly turned outwards, so as to afford a hold for the fingers for pulling the tube out. The tube so constructed not inaptly reminds one of Recamier's speculum, and might, on occasion, be employed as such, as I have done.

A sponge of the finest texture, and oval, about the size of a pullet's egg (when dry), is firmly sewn on a round and slender ivory washer, half an inch in diameter, perforated with holes for the needle to pass through, and having moreover a female screw in the centre, into which the ivory stem is screwed when the instrument is to be introduced. This arrangement allows the same stem being employed with two, three, or more sponges similarly secured on their respective ivory washers, whereby great facility is afforded of changing the pessary daily or more frequently, for the sake of cleanliness and comfort. The sponge and the stem thus combined constitute the pessary, the introduction of which by the patient herself is effected in the following manner:—

The stem, bearing the sponge, being introduced by its free end into the upper or narrower part of the tube, is gradually pushed down towards the wider or lower end until the sponge itself disappears almost entirely, leaving only a very small, round segment visible. By moistening the sponge and compressing it, this manœuvre becomes easy of accomplishment. The patient next separates the labia with her left hand, and introduces with the other the instrument by its smaller end into the vagina, either in a standing or sitting posture, pushing it first somewhat backward and then upwards, until the everted edges touch the labia. In this position she applies one or two fingers to the small, round knob which terminates the stem, and holds it firm in its position; while with the fingers of the right hand she withdraws, by means of the everted edges, the tube from the vagina—an operation greatly facilitated by the presence of the two semi-oval cuts. The pessary will now remain in its place without wabbling or falling; but it may further be secured, if so desired (though there is little occasion for it,) by passing a staylace through a hole made for that purpose in the small end knob of the stem, and fastening the same to a belt or a napkin.

To withdraw the instrument the patient, standing and bending forward, has only to pull the stem by the knob with the staylace, or even without it, at first straight downwards, and next by bringing the stem more towards the abdomen. In every case in which I recommended such a contrivance among patients capable, from their position in society, to appreciate the boon, the result has been most successful. The feeling of confidence and support experienced by the patient has been universally acknowledged. Mr. Weiss, instrument-maker, to whom I made over one of the instruments manufactured under my direction for the information of the profession, has kindly undertaken to have others made after the pattern, and keep them ready for use. I remain, Sir, your obedient servant,

A. B. Granville, M.D., F.R.S.

*Curzon-street, May-fair, July,* 1862.

---

### THE TREATMENT OF ANEURISM.

To the Editor of the Lancet:—

Sir:—Since much important information relative to the treatment of aneurism has appeared in the *Lancet*, may I make so bold as to request you, should you think it worthy a place in your journal, to insert the following suggestion relative to the treatment of aneurism—a suggestion which I trust may lead in many cases to the abandonment of ligature, and fill up the gap which appears to me to exist between the simpler forms of treatment, such as by pressure and forcible flexion, and the extreme one by ligature—viz.: to cut down upon the artery, and to so far separate its coats from the surrounding structures as to enable the operator to control the circulation through the vessel by a small pair of compress forceps, electro-plated, with a good firm spring, and slightly roughened on their approximate surfaces so as to prevent them slipping from the vessel. By this means the circulation might be as effectualy stopped as by the ligature, and the necessity of separating the vein from the artery (which in cases of the femoral artery is the chief obstacle to the operation) done away with. The forceps should be left in the wound, and secured by plaster, till the cure is effected. I do not think any more serious consequences would follow such a proceeding than would follow the ligature, and it would have the advantages—1st, facility in the operation; 2ndly, avoid the separation of the vein; 3rdly, the forceps could be removed at any moment, should it be found necessary to do so. In those cases of internal piles, where their removal by the knife is prevented by the fear of subsequent hemorrhage, I am of opinion that the compress forceps applied to the base of the pile might be used instead of the ligature to restrain the bleeding, and could be removed after ten, twelve, or more hours after the operation, without any fear of bad results.

I am very glad to see in one of your late numbers a proposal for the removal of embolon. Perhaps I may be allowed to state that I have held the opinion that a fine suture might be applied to the coats of both arteries and large veins in cases of accidental wounding during operation, or from other accidental causes, such as puncture by penknife, etc. We cannot possibly know how an operation may succeed until it is tried. Should a case of aneurism come under my notice, and the simpler methods fail to afford a cure, I shall attempt its cure by the compress forceps.

Hoping that I have not suggested an impracticable operation, and with the desire to supply a link in the chain of operations for aneurism. I am Sir, your obedient servant,

AUGUSTUS BROWN, M.R.C.S.

Minerva-terrace, Barnsbury-park, Islington, June, 1862.

---

THE POISON OF THE ADDER.—Dr. Edwin Bishop, of Moreton Hampstead, Devon, in a letter to *The Times*, makes the following suggestion:—

"The remedy for the bite of an adder, and indeed all poisonous snakes, is simple. It has been proved by experiment, over and over again, that poisons of this character are harmless when applied to a mucous service, and quantities have been swallowed without producing any ill-effect. The bite, nine times out of ten, is in some part of the hand, and immediately it is felt the wounded part should be well sucked by the mouth, and a piece of string tied tight round the finger or the wrist (according to the seat of the bite) to prevent the poison from being absorbed into the system. If this simple plan was generally known and acted upon, death or injury from the bite of an adder would be rare indeed."

In cases of dissecting wounds, and of hydrophobia, this plan might also be adopted, and in many instances would prevent the poison entering the system.

---

ACETATE OF POTASH IN GONORRHŒA.—Dr. Betoldi states in the May number of the *Annali di Medicina*, that he has had much success with this salt in gonorrhœa, given in doses of about one drachm a day. The author seems to look upon this medication as novel, and is probably not aware that acetate of potash has for some time been given in this country in cases of gonorrhœa, with variable results.

---

A HUMAN SALAMANDER.—A curious exhibition takes place every evening at the corner of the Rue Ville Just and the Avenue de St. Cloud, Paris. In a small field there situated a wooden house, covered with pitch and other combustible matters, is erected daily, and set fire to at about eight o'clock each evening. A man jumps into the midst and pulls down blazing rafters, which he carries away. This salamander can stay in the fire from five to seven minutes. His clothes are said to be made of asbestos, covered with sponges freely imbued with some chemical preparation.

---

OPHTHALMIA.—The new species of ophthalmia which startled the French physicians a few weeks ago in the garrison of Vincennes, is more extensively spread than was at first supposed. The soldiers whose optic nerves become paralysed the moment the sun goes down are not to be found in that locality alone. The disease has appeared with great virulence at Strasburg and other places, and has much embarrassed the faculty. Perfect vision returns in the morning, but at sunset the patients again lose their sight. The most searching investigation is being made as to its cause and cure.

THE HAIRLESS MEN OF AUSTRALIA.—Their exists beyond the Balonne River, Western Australia, a race of men entirely destitute of hair on any part of their bodies. They appear to be of Mongolian or Chinese origin.

TEST FOR POISONOUS PAPER-HANGINGS.—Common spirits of hartshorn or armmonia is a very sure and easy test for arsenic. On application, the beautiful but dangerous green turns to a decided blue.

*L' Union Medicale* gives the following catalogue of diseases under which the Grand Monarch, Louis XIV., suffered at various times of his life. The facts are obtained from observations made by his physicians, Vallot, D'Aquin, and Fagon. The king appears to have had his share of human bodily infirmities. "At seventeen, he caught a violent blennorrhœa. Some time afterwards, he narrowly escaped falling a victim to confluent small-pox. He next suffered from a scirrhous induration of the breast. At Calais he caught a putrid fever; and afterwards had the measles, dysentery, odontalgia, arthritis, abscess in the left armpit, and fistula in ano. Besides this, Louis XIV. had an anthrax in the neck. Moreover, all the teeth in his upper jaw were extracted, in consequence of a fistulous opening in this bone, which made a communication between the mouth and the nose, so that liquids escaped by the nose, the royal nasal mucosities giving off a cadaverous odor. Lastly, he is represented as having been bled forty-four times, as having had fifty lavements, and swallowed two hundred and eleven purgatives. Gouty, podagrous, and afflicted with gravel during many years, this lofty individual at last perished of gangrena senilis."—*Brit. Med. Journal.*

BRIGADE-SURGEON ADAM HAMMER is ordered to report to the Medical Director at St. Louis for duty in charge of one of the general hospitals in that city.

Assistant-Surgeon Harrison Allen, Medical Cadet F. G. H. Bradford, and Hospital Stewards McManus and Austen will report in person to Surgeon Letterman, Medical Director of the Army of the Potomac, for duty.

Surgeon Mitchell, 1st Maryland Volunteers, will report for duty to Surgeon McVarlin, Menical Director of Gen. Pope's command.

Surgeon Parker, U. S. Army, will repair to Chicago to relieve Brigade-Surgeon Blaney, in his duties as Medical Purveyor, the latter to report to the Surgeon-General.

Surgeon William Whela, to be Chief of the Bureau of Medicine and Surgery.

Theoron Woolverton, of New York, to be Assistant-Surgeon in the Navy.

J. H. Boucher, of Iowa, to be Brigade-Surgeon of Volunteers.

The following were confirmed as Assistant-Surgeons in the United States Army:—Wm. H. Keene, of Pennsylvania, Geo. L. Porter, of Pennsylvania, David S. Huntington, of Pennsylvania, T. W. Williams, District of Columbia, Charles M. Colton, of Virginia, T. M. Brown, of Ohio, Charles S. Degraw, of New York, Edward C. Strode, of Illinois, Andrew H. Smith, of New York, and Van Buren Hubbard, of Ohio.

Brigade-Surgeon Lecompte has been ordered to repair to Chester, Penn., to take charge of the general hospital at that place.

Brigade-Surgeon C. L. Allen is ordered to report to the Surgeon-General as a member of the Board for the Examination of Surgeons of Volunteers.

Brigade-Surgeon A. B. Crosby, late Medical Director Peck's Division, one of the most talented and energetic medical officers in the army, has resigned, and will return home immediately. Ira Perry, Contract-Surgeon, has been assigned to the 2d R. I., as Act.-Asst.-Surgeon. J. G. Strowbridge to the 39th Ill.; J. W. Hinchley to the 13th Ind. Drs. Sargent, M'Collister, and Pierce to the batteries under command of Major West.

Brigade-Surgeon D. H. Prince and Surgeon H. Jewitt, of the 10th Mass., remained with the wounded after the battle of Malvern, are still in the hands of the enemy. Surgeon M. S. Killenger of the 100th N. Y. remained with his wounded on the Chickahomony, and is a prisoner.—*Am. Med. Times.*

